# Editorial: Brain metabolic imaging by magnetic resonance imaging and spectroscopy: methods and clinical applications

**DOI:** 10.3389/fnins.2023.1239243

**Published:** 2023-06-23

**Authors:** Jiazheng Wang, Liangjie Lin, Tao Gong, Zhiliang Wei, Yong Zhang

**Affiliations:** ^1^Clinical and Technical Support, Philips Healthcare, Beijing, China; ^2^Department of Radiology, Shandong Provincial Hospital Affiliated to Shandong First Medical University, Jinan, China; ^3^Department of Radiology, Shandong Provincial Hospital, Cheeloo College of Medicine, Shandong University, Jinan, China; ^4^The Russell H. Morgan Department of Radiology and Radiological Science, Johns Hopkins University School of Medicine, Baltimore, MD, United States; ^5^F. M. Kirby Research Center for Functional Brain Imaging, Kennedy Krieger Research Institute, Baltimore, MD, United States; ^6^Department of Magnetic Resonance Imaging, The First Affiliated Hospital of Zhengzhou University, Zhengzhou, China

**Keywords:** MRI, MRS, metabolic imaging, central nervous system, clinical translation

The past decade has witnessed the fast development of precise medicine in a wide spectrum of diseases, driven by the progress of targeted therapy and immunotherapy. For example, the clinical translation of proteolysis-targeting chimera (PROTAC) has enabled targeting on those targets that were previously considered “undruggable” (Bekes et al., 2022). Antibody-drug conjugates have demonstrated clear overall survival (OS) benefits in selected cancer patients by linking cytotoxic agents to antibodies specifically targeting tumor or tumor-associated cells (Thomas et al., 2016). The multifaceted efforts in exploring the PD-1 inhibitory pathway have resulted in both the widespread adoption of PD-1/PD-L1 inhibitors for cancerous diseases and the recent evidence in PD-1 pathway stimulation for autoimmune diseases (Tuttle et al., 2023). However, conventional radiological response criteria, such as response evaluation criteria in solid tumors (RECIST), are still generally practiced to assess these precise treatments, while these criteria are considered inadequate in accountable for the changes in the pathological tissues induced by the novel treatments (Shankar et al., 2023). Therefore, there is a clear need in the clinic for medical imaging methods that measure the cellular pathological process more directly. In this Research Topic “*Brain metabolic imaging by magnetic resonance imaging and spectroscopy: methods and clinical applications*,” researchers have demonstrated the potential of metabolic imaging with the clinical setup in a range of different central nervous system (CNS) diseases.

It is widely anticipated that metabolic alterations precede structural changes in many CNS diseases. Chronic insomnia (CI) is associated with increased risk of infections and all-cause mortality, for which Chen W. et al. observed increased instability in the dynamic local brain activity in the CI patients, by interpreting the functional MRI study using the dynamic fractional amplitude of low-frequency fluctuation (dfALFF) analysis. On the neurodegenerative disease, Chen X. et al. detected lower glutamate-glutamine (Glx) signal and higher amide proton transfer-weighted (APTw) signal in the right hippocampus in patients with amnestic mild cognitive impairment (aMCI). To distinguish the aMCI patients from the health control, the area under the curve (AUC) was 0.88 in the ROC curve analysis by combining these two parameters (sensitivity 0.7, specificity 0.95), potentially facilitating the medical interventions to delay the onset of Alzheimer's disease. While the connection between CNS disease and neurofilament light chain (NfL) has become an active research field in the past a few years, Huang et al. revealed positive correlation between the APTw signal and the serum NfL concentration in multiple sclerosis (MS) patients, likely suggesting the association between elevated APTw signal and the axonal injury. Significant metabolic abnormalities were found by Salan et al. in perinatally HIV (PHIV) infected young adults, in parallel to the reduction in CD4+ T cell count, and were in agreement with the long-term brain structural and functional deficiencies in PHIV patients.

On the other hand, metabolic changes could also happen after brain injury and might be associated with neurological outcomes. Robayo et al. observed decreased N-acetylaspartate (NAA), total creatine, and total choline in pain-related brain regions in patients with chronic post-traumatic pain. These changes were considered as the result of disrupted metabolic and structural integrity in the brain. Wu et al. provided a systematic review on the recent progress of MRI in the diagnosis of sepsis-associated encephalopathy (SAE) in post-traumatic patients, for which metabolic MRI were shown with the potential to provide effective imaging biomarkers such as decrease in NAA (indicating neuronal loss or dysfunction) and in choline (indicating cell death). Other MR-based functional evaluations such as arterial spin labeling (ASL) and diffusion-weighted imaging (DWI) were also reviewed.

While detecting distant metastasis has remained the dominating clinical scenario for ^18^F-FDG PET, Zhang et al. has demonstrated their preliminary experience combining ^18^F-FDG PET and MRI in the distinguishment of instable carotid plaques. In a hybrid setup, the ^18^F-FDG PET was able to evaluate the inflammatory condition in the plaque, depending on the ^18^F-FDG uptake by the macrophages, and the high-resolution MRI was able to provide morphological information on the plaque content. It would be very interesting if some more recent and more advanced MRI techniques, such as the simultaneous non-contrast angiography and intraplaque hemorrhage (SNAP) imaging (Jia et al., 2022), could be included in the study. Nevertheless, this work has exemplified the coordinated implementation of MRI and nuclear medicine, which is probably necessary to account for the multimodal nature of the complicated and systemic diseases (Hricak et al., 2021).

With association studies performed and diagnosis model established, these works have demonstrated the value of metabolic imaging in the diagnosis, the monitoring, and the treatment guidance of a spectrum of CNS diseases. There remain several unanswered questions before the clinical translation of these methods. For example, as frequently criticized for its lack of clinical success, metabolic MR techniques need to be examined in larger cohorts, where higher level of clinical evidence could be generated. To accomplish this, appropriate study endpoints should be selected with cautiousness, and quality control should be performed to adjust for the across-site and across-vendor variations (Wang and Hesketh, 2023). On the other hand, the recent progress on artificial intelligence (AI) may shed light on more accurate and faster quantification of MR-based metabolic information, and the use of hyperpolarized MR techniques could dramatically enhance the *in vivo* sensitivity of the metabolites. Together with other increasingly practiced studies such as CEST and deuterium imaging, the next decade may see an increasing trend of clinical translation of metabolic MRI, particularly with the collective information acquired on a range of different nuclei—the concept of MR Nucleomics (Sun et al., 2023).

## Author contributions

Supervision: YZ. Writing—original draft: JW. Writing—review and editing: LL, TG, ZW, and YZ. All authors contributed to the article and approved the submitted version.
